# The Resistance Mechanisms and Clinical Impact of Resistance to the Third Generation Cephalosporins in Species of *Enterobacter cloacae* Complex in Taiwan

**DOI:** 10.3390/antibiotics11091153

**Published:** 2022-08-26

**Authors:** Chung-Yu Chang, Po-Hao Huang, Po-Liang Lu

**Affiliations:** 1 School of Post-Baccalaureate Medicine, College of Medicine, Kaohsiung Medical University, Kaohsiung 80708, Taiwan; 2 Department of Microbiology and Immunology, School of Medicine, College of Medicine, Kaohsiung Medical University, Kaohsiung 80708, Taiwan; 3 Graduate Institute of Medicine, College of Medicine, Kaohsiung Medical University, Kaohsiung 80708, Taiwan; 4 Division of Infectious Diseases, Department of Internal Medicine, Kaohsiung Medical University Hospital, Kaohsiung Medical University, Kaohsiung 80708, Taiwan; 5 Center for Liquid Biopsy and Cohort Research, Kaohsiung Medical University, Kaohsiung 80708, Taiwan; 6 Department of Biological Science and Technology, College of Biological Science and Technology, National Yang Ming Chiao Tung University, Hsinchu 30068, Taiwan; 7 M.Sc. Program in Tropical Medicine, College of Medicine, Kaohsiung Medical University, Kaohsiung 80708, Taiwan

**Keywords:** *Enterobacter*, *hsp60* gene, mortality, cluster

## Abstract

*Enterobacter cloacae* complex (ECC) is ubiquitous in the environment and is an important pathogen causing nosocomial infections. Because routine methods used in clinical laboratories cannot identify species within ECC, the clinical significance of each species within ECC is less known. We applied *hsp60* gene sequencing to identify the species/clusters of ECC and detected β-lactamase genes and class 1 integrons with PCR for 184 clinical ECC isolates in Taiwan from 2013 to 2014 to investigate the clinical impact of species within ECC. The four most common clusters were *E. hormaechei* subsp. *steigerwaltii* (cluster VIII) (29.9%), *E. hormaechei* subsp. *oharae* (cluster VI) (20.1%), *E. cloacae* subsp. *cloacae* (cluster XI) (12%), and *E. kobei* (cluster II) (10.3%). *E. hormaechei*, which consisted of four clusters (clusters III, VI, VII, and VIII), is the predominant species and accounted for 57.1% of the isolates. The ceftazidime resistance rate was 27.2%, and the ceftriaxone resistance rate was 29.3%. Resistance to third generation cephalosporin was associated with a higher 30-day mortality rate. In total, 5 (2.7%), 24 (13.0%), and 1 (0.5%) isolates carried ESBL, AmpC, and carbapenemase genes, respectively. Class 1 integrons were present in 24.5% of the isolates, and most of the cassettes pertain to antibiotic resistance. Resistance to third generation cephalosporins, multidrug resistance, and class 1 integrons were significantly more in *E. hormaechei* (clusters III, VI, VII, and VIII) than in the other species. The 30-day mortality rate and 100-day mortality did not differ significantly between patients with *E. hormaechei* and those with infections with the other species. In conclusion, the distribution of third generation cephalosporin resistance, multidrug resistance, and class 1 integrons were uneven among *Enterobacter* species. The resistance to third generation cephalosporins possessed significant impact on patient outcome.

## 1. Introduction

*Enterobacter cloacae* complex (ECC), which belongs to the family *Enterobacteriaceae*, is ubiquitous in various environments. *Enterobacter* is an important pathogen causing nosocomial infections [1,2]. There are seven ECC species: *E. cloacae*, *E. hormaechei*, *E. asburiae*, *E. kobei*, *E. ludwigii*, *E. nimipressuralis*, and *E. mori* [3]. To date, there are 22 species with updated nomenclature in the *Enterobacter* genus according to new data and analysis results from whole genome sequencing (https://lpsn.dsmz.de/genus/enterobacter, accessed on 10 July 2022) [3,4]. However, routine biochemical and phenotypic methods employed by clinical laboratories are unable to completely distinguish the species of ECC [5,6]. Molecular and genomic approaches have been applied for EC species identification, including heat-shock protein 60 (*hsp*60) typing, multilocus sequence typing (MLST), and whole-genome sequencing (WGS) [7]. By sequencing the *hsp60* gene, Hoffman and Roggenkamp divided ECC into 13 genetic clusters (clusters I to XII and cluster XIII, which corresponds to an unstable sequence crowd based on the *k* parameters) [8]. The MLST scheme has emerged as a robust tool for identifying closely related *Enterobacter* species [9]. More than 1900 sequence types have been reported in the MLST database (https://pubmlst.org/organisms/enterobacter-cloacae/, accessed on 10 July 2022). WGS provides the opportunity to explore the genetic relationships between genomes, and the use of WGS revealed 22 phylogenetic clades (A–V) [7,10,11,12].

The antimicrobial resistance issue of ECC raised clinicians’ concern [1]. ECC isolates are intrinsically resistant to ampicillin, amoxicillin–clavulanate, and first and second generation cephalosporins because they express intrinsic AmpC β-lactamases, i.e., CMH, ACT, and MIR with multiple variants [11,13,14]. Owing to the widespread use of antibiotics, multidrug resistant (MDR) ECC strains have emerged and spread globally [7,15]. AmpC overproduction is usually associated with resistance to most of the third generation cephalosporins [16]. The acquisition of genes encoding extended spectrum β-lactamase (ESBL) also leads to resistance to the third generation cephalosporins [11,16]. Moreover, the emergence and increasing prevalence of carbapenem-resistant ECC is causing treatment difficulty [17]. Infections caused by MDR strains usually result in higher mortality, longer hospitalizations, and higher costs, thus exerting immense impact on global public health [18,19].

According to previous reports from Taiwan, the prevalence of ESBL-producing *E. cloacae* is in the range of 15%–28%, and the main ESBL genotype is *bla*_SHV_ [20], while *bla*_CTX-M_ [21,22,23] also exists. AmpC β-lactamase genes of ECC include intrinsic *bla*_MIR_ and *bla*_ACT_ as well as acquired *bla*_DHA_ and *bla*_CMY_ [21]. The results from the Study for Monitoring Antimicrobial Resistance Trends in Taiwan between 2016 and 2018 revealed that isolates of *Enterobacter* species showed higher rates of ESBL and nonsusceptibility to ertapenem than those of *Escherichia coli* or *Klebsiella pneumoniae* isolates [24]. In our previous study on carbapenem-nonsusceptible *E. cloacae* complex infections [25], *bla*_IMP-8_ was the only detected carbapenemase gene, which was consistent with previous Taiwanese studies [21,22]. In addition, most studies in Taiwan on antimicrobial resistance and clinical infection refer to “*E. cloacae*”, and there has been no investigation on whether there are differences between ECC species/clusters. In this study, we collected *Enterobacter* isolates identified as *E. cloacae* complex using a VITEK2 automated identification system in a clinical bacteriology laboratory. Hence, we aimed to explore the distribution of species/cluster, clonal relatedness, antimicrobial resistance, β-lactamase genes, and clinical features of ECC isolates in Taiwan.

## 2. Results

### 2.1. Species Identification of ECC Isolates Based on hsp60 Sequencing

A total of 184 ECC isolates were included in the study. One hundred and eighty of the isolates (97.8%) were classified into 14 species/subspecies (10 clusters) using *hsp60* gene sequencing (Table 1). The four most common clusters were *E. hormaechei* subsp. *steigerwaltii* (cluster VIII) (55/184, 29.9%), *E. hormaechei* subsp. *oharae* (cluster VI) (37/184, 20.1%), *E. cloacae* subsp. *cloacae* (cluster XI) (22/184, 12%), and *E. kobei* (cluster II) (19/184, 10.3%). However, the most common species was *E. hormaechei* (105/184, 57.1%), which consisted of four clusters (clusters III, VI, VII and VIII). There were three species other than previously defined ECC species and clusters: *E. chuandaensis* (1 isolate), *E. quasihormaechei* (2 isolates), and *E. sichuanensis* (1 isolate). Four isolates (2.2%) not assigned to any species/clusters were classified as “not determined”. Wu et al. proposed an updated classification and nomenclature of the genus *Enterobacter* in recent years [4], and the corresponding species are listed in Table 1. Figure S1 (Supplementary File S1) shows the phylogenetic tree resulting from analysis of the *hsp60* gene sequences of 184 *Enterobacter* isolates and the sequences of type strains. The partial *hsp60* sequences of 184 *Enterobacter* isolates are listed in Supplementary File S2.

### 2.2. The Distributions of the Isolation Sites of Enterobacter

The most common isolation specimen was urine (54/184, 29.3%), followed by sputum (47/184, 25.5%), abscess/pus (38/184, 20.7%), and blood (27/184, 14.7%). No significant difference for the specimen distribution was found between species/clusters.

### 2.3. Antimicrobial Susceptibility of Enterobacter Species

The antimicrobial resistance rates for each species of the 184 *Enterobacter* isolates are shown in Table 2. All the *Enterobacter* isolates were susceptible to amikacin. Additionally, more than 90% of *Enterobacter* showed susceptibilities to meropenem (99.5%), cefepime (98.9%), ertapenem (95.7%), gentamicin (92.9%), levofloxacin (92.9%), and tigecycline (92.4%). Fifty strains (27.2%) were resistant to ceftazidime and 54 strains (29.3%) were resistant to ceftriaxone. Among the 50 ceftazidime-resistant isolates, 47 were resistant to ceftriaxone, two were intermediate susceptible, and one was susceptible to ceftriaxone. In addition, Table 2 shows the resistance of various species to third generation cephalosporins, of which *E. sichuanensis*, *E. hormaechei* subsp. *oharae* (cluster VI), *E. hormaechei* subsp. *steigerwaltii* (cluster VIII), and *E. hormaechei* subsp. *hoffmannii* (cluster III) had a higher resistance rate to third generation cephalosporins than other species, i.e., 100% (1/1), 40.5% (15/37), 30.9% (17/55), and 30.0% (3/10), respectively for ceftazidime; 100% (1/1), 40.5% (15/37), 34.5% (19/55), and 50% (5/10), respectively for ceftriaxone. *E. hormaechei* subsp. *hormaechei* (cluster VII) also presents high rates of ceftazidime resistance (33.3%, 1/3). Among ceftazidime- and ceftriaxone-resistant strains, *E. hormaechei* (clusters III, VI, VII and VIII) accounted for 72% (36/50 for ceftazidime and 39/54 for ceftriaxone) of the isolates. Moreover, the resistance rates to third generation cephalosporins in *E. hormaechei* (clusters III, VI, VII, and VIII) were significantly more than those in the other species (36 isolates for ceftazidime and 39 isolates for ceftriaxone vs. 14 isolates for ceftazidime and 15 isolates for ceftriaxone, both *p* < 0.05).

Twenty-three (12.5%) of the 184 *Enterobacter* isolates showed multidrug resistance. *E. hormaechei* subsp. *hoffmannii* had the highest percentage (80%, 8 strains) of multidrug resistance, followed by *E. hormaechei* subsp. *hormaechei* (33.3%, 1 strain), *E. hormaechei* subsp. *oharae* (16.2%, 6 strains), *E. hormaechei* subsp. *steigerwaltii* (12.7%, 7 strains), and *E. cloacae* subsp. *cloacae* (4.5%, 1 strain). Multidrug resistance was significantly more present in *E. hormaechei* than in other species (*p* < 0.001).

### 2.4. β-Lactamase Genes of Enterobacter Isolates

The presence of genes encoding β-lactamases is summarized in Table 3. It was found that 5 (2.7%), 24 (13.0%), and 1 (0.5%) isolates carried ESBL, AmpC, and carbapenemase genes, respectively. The ESBL genes included *bla*_SHV-12_ (4 isolates) and *bla*_CTX-M-15_ (1 isolate). Among 24 *bla*_AmpC_-positive *Enterobacter* isolates, genes encoding ACT, DHA-1, and MIR accounted for 83.3% (20 isolates), 25% (6 isolates), and 12.5% (3 isolates), respectively. The carbapenemase gene in one isolate was *bla*_IMP-8_. Co-carriage of *bla*_ACT_ and *bla*_DHA-1_ was found in five isolates, of which two also carried *bla*_SHV-12_.

### 2.5. Class 1 Integrons and Gene Cassettes in Enterobacter Isolates

Class 1 integrons were present in 45 isolates (24.5%, 45/184), and the cassette regions were amplified from 32 of the 45 isolates (Table 4). The gene cassette arrays of these class 1 integrons for each *Enterobacter* species are listed in Table 4. Most of the cassettes pertained to antibiotic resistance genes, including those encoding resistance to trimethoprim (*dfrA7*, *dfrA12*, *dfrA15*, and *dfrA27*), gentamicin (*aadB*), streptomycin (*aadA1*, *2*), rifampin (*arr*3), aminoglycosides (*aac*3 and *aac(6′)-IIc*), and/or decreased fluoroquinolone susceptibility (*aac(6′)-Ib-cr*). We found the erythromycin gene *ereA2* to be functionless because it is disrupted. A 6.2 kb long cassettes region containing the gene cassettes *aac(6′)IIc-ereA2-*IS*1247-aac3-arr-ereA2* was present in one *E. hormaechei* subsp. *hoffmannii* and one *E. hormaechei* subsp*. oharae*. In addition, five isolates carried two or more class 1 integrons within a single strain (four isolates carried two class 1 integrons and one isolate carried three class 1 integrons). More *E. hormaechei* subsp*. oharae* strains contained class 1 integrons than *E. kobei* and *E. hormaechei* subsp. *steigerwaltii* (cluster VI vs. II, *p* = 0.028; cluster VI vs. VIII, *p* = 0.004). Class 1 integrons were significantly more present in *E. hormaechei* (34.3%) than in other clusters (11.4%, *p* < 0.001).

### 2.6. PFGE Analysis

The PFGE analysis revealed 176 pulsotypes among the 184 isolates. Only 5 pulsotypes contained more than one isolate. The 5 pulsotypes were from 13 (7.1%, 13/184) isolates. Two pulsotypes contained 4 and 2 isolates of *E. hormaechei* subsp. *hoffmannii* (cluster III), respectively. One pulsotype consisted of 1 *E. cloacae* subsp. *cloacae* isolate and 1 “not determined” isolate. The other two pulsotypes contained 3 *E. hormaechei* subsp*. oharae* (cluster VI) isolates and 2 *E. hormaechei* subsp. *steigerwaltii* (cluster VIII) isolates, respectively.

### 2.7. Clinical Features of Patients Infected with Enterobacter

The above results showed that resistance-associated characteristics such as third generation cephalosporin resistance and class 1 integrons were mostly present in *E. hormaechei* (clusters III, VI, VII, and VIII). Therefore, we further examined if there was any difference in clinical characteristics between infection with *E. hormaechei* (clusters III, VI, VII, and VIII) and other species/clusters of *Enterobacter*. Table S1 (Supplementary File S3) revealed that the main differences between these two groups were antimicrobial resistance-related factors, such as third generation cephalosporin resistance and class 1 integrons. The proportion of patients infected with *E. hormaechei* was lower than those infected with other clusters of *Enterobacter* for healthcare-associated infection and related to surgery. There were no statistically significant differences in the other demographic data, comorbidities, therapeutic devices and procedures, and clinical outcomes (30-day and 100-day mortality).

The clinical features and significance of susceptibility to third generation cephalosporins in *Enterobacter* are summarized in Table 5. Patients with *Enterobacter* resistant to third generation cephalosporins were significantly associated with higher percentages of underlying diseases of kidney disease, indwelling devices use, ICU admission, and class 1 integrons. Moreover, patients with *Enterobacter* resistant to the third generation cephalosporins were more likely to have a significantly higher 30-day mortality (OR: 6; 95% CI: 2.24–16.06) and 100-day mortality (OR: 5.74; 95% CI: 2.24–14.70) than those infected with *Enterobacter* susceptible/intermediate to third generation cephalosporins. Furthermore, we analyzed the clinical characteristics of *Enterobacter* infection caused by the four most common species/clusters in this study (*E. hormaechei* subsp. *steigerwaltii*, *E. hormaechei* subsp*. oharae*, *E. cloacae* subsp. *cloacae*, and *E. kobei*; clusters VIII, VI, XI, and II). Table S2 (Supplementary File S3) shows that there were significant differences in the clinical characteristics of the four clusters, which included gastrointestinal disease (*p* = 0.042), hemodialysis (*p* = 0.020), site of acquisition (hospital-acquired and community-acquired, *p* = 0.018), class 1 integrons (*p* = 0.010), and outcomes (30-day mortality, *p* = 0.016; 100-day mortality, *p* = 0.014). *E. cloacae* subsp. *cloacae* (cluster XI) occurred more frequently than the other three species in community-acquired infections (38.9%). In addition, the proportion of third generation cephalosporin resistant *E. hormaechei* subsp*. oharae* (cluster VI) strains was significantly higher than that of *E. kobei* (cluster II) (cluster VI vs. II, *p* = 0.028). A higher proportion of patients infected with *E. cloacae* subsp. *cloacae* (cluster XI) had poor outcomes in terms of 30-day mortality (XI, 33.3%; II, 0; VI, 18.2%; VIII, 8.5%) and 100-day mortality (XI, 33.3%; II, 0; VI, 21.2%; VIII, 8.5%) than those infected with the other three species.

## 3. Discussion

We aimed to investigate the clinical and microbiological characteristics of the species within *E. cloacae* complex (ECC) in this research. Our bacteria material was ECC isolates which were routinely identified from the clinical laboratory. Of the 184 isolates, 97.8% were classified into species and clusters based on *hsp60* sequencing. However, three species other than previously defined ECC species and clusters were identified. It revealed the limitation of *Enterobacter* species identification with *hsp60* sequencing. It was reported that determining taxonomic assignment using a single-gene-based approach may miss valuable information available from the rest of the genome and potentially lead to unreliable conclusions about taxonomic positions [4]. Given that the taxonomy of *Enterobacter* is complicated, we have added the nomenclature information with an updated classification and nomenclature of the genus *Enterobacter* using genome sequence-based analysis [4] for our isolates in Table 1.

The molecular epidemiology via PFGE revealed no large outbreak of *Enterobacter* due to specific clones in the Taiwan medical center. Under this background, we identified that the most common identified species/clusters in Taiwan are *E. hormaechei* subsp. *steigerwaltii* (cluster VIII) (29.9%), followed by *E. hormaechei* subsp. *oharae* (cluster VI) (20.1%), *E. cloacae* subsp. *cloacae* (cluster XI) (12%), and *E. kobei* (cluster II) (10.3%). Most other data for species distribution in ECC are from European countries. We summarize the distribution of different ECC species in different countries in Table S3 (Supplementary File S3). *E. hormaechei* subsp. *steigerwaltii* (cluster VIII) and *E. hormaechei* subsp. *hoffmannii* (cluster III) were the two most common clusters in Europe [5,6,26]. However, clusters VI and VIII accounted for most of the Taiwan isolates (50%), whereas *E. hormaechei* subsp. *hoffmannii* (cluster III) only accounted for 5.4%. This cluster distribution is similar to that in Guadeloupe where clusters VI and VIII accounted for 56.1% and cluster III was rare [11]. Though *E. hormaechei* subsp. *hoffmannii* (cluster III) was not common in clinical *Enterobacter* isolates in Taiwan, it was noteworthy that it is the most commonly identified species among carbapenem-nonsusceptible *E. cloacae* complex in Taiwan and in Southeast China [15,25]. Furthermore, clusters VI and VIII belong to a species named *E. xiangfangensis* as recommended by Wu et al. [4]. Most (63.5%) *Enterobacter* strains from human bloodstream infection in China are *E. xiangfangensis* [27]. According to the new nomenclature system, this species was also the most common *Enterobacter* species in our clinical isolates.

The lower rates of antibiotic resistance were observed among clinical *Enterobacter* isolates from Taiwan compared to those reported from Poland and Guadeloupe, including: amikacin (0% vs. 56.5% vs. 1%), ceftazidime (27.2% vs. 55.7% vs. 56.1%), gentamicin (7.1% vs. 55.1% vs. 22.4%), and sulfamethoxazole/trimethoprim (18.5% vs. 55.1% vs. 38.3) [11,28]. More than 70% of *Enterobacter* isolates resistant to third generation cephalosporins belonged to the four *E. hormaechei* clusters (III, VI, VII, and VIII). We observed similar findings with data from France and Guadeloupe in that *E. hormaechei* carried higher resistance rates to third generation cephalosporins when compared with other *Enterobacter* clusters [11,16].

In the study, only 2.7%, 13.0%%, and 0.5% of the *Enterobacter* isolates carried *bla*_ESBL_, *bla*_AmpC_, and carbapenemase genes, respectively (Table 3). The percentages of *bla*_ESBL_ and carbapenemase genes among *Enterobacter* strains from Taiwan (2.7% and 0.5%, respectively) were slightly lower than those from the United States (3% and 3%) [29], but far lower than those from Nepal (80.3% and 59.6%) [30]. That the rates with β-lactamase genes were lower than the resistance rate to third generation cephalosporins signifies that β-lactamase production partially contributed to the resistance to third generation cephalosporins and there may be other mechanisms of resistance to third generation cephalosporins such as efflux pumps, reduced permeability, and altered transpeptidases [31].

Class 1 integrons were found in 24.5% of the *Enterobacter* isolates, whereas 55% of the *Enterobacter* isolates in Poland carried class 1 integrons [28], which might be associated with the difference of antimicrobial resistance rates of *Enterobacter* in the two countries. In our surveillance, class 1 integrons are mostly distributed in three *E. hormaechei* clusters (clusters VI, III, and VIII). In Poland, class 1 integrons were found mostly in *E. hormaechei* subsp. *steigerwaltii* (cluster VIII), accounting for 81.6% of class 1 integron-positive strains [28]. In this study, resistant gene cassettes carried on class 1 integrons, such as *dfrA*, *aadA*, and *aadB*, were widespread in class 1 integrons, which agrees with previous studies [32,33,34,35].

For effective treatment of *Enterobacter* infection, the best options among the β-lactams are the fourth generation cephalosporins (e.g., cefepime and cefpirome) and carbapenems in the literature. The aminoglycosides (particularly amikacin) also have a good activity [3]. Our findings of the antimicrobial susceptibility to *Enterobacter* agrees with the literature report. To counteract β-lactamases, piperacillin-tazobactam has been found to be a valuable treatment option for *Enterobacter* spp. bloodstream infections [3,36]. Newer β-lactam/β-lactamase inhibitor combinations (cefepime-zidebactam, cefepime-tazobactam, ceftolozane-tazobactam, ceftazidime-avibactam, meropenem-vaborbactam, imipenem-relebactam, etc.) are now available for clinical use [3,37]. Moreover, the combinations polymyxin B/amikacin, polymyxin B/tigecycline, and polymyxin B/meropenem are promising for treatment of carbapenem-resistant *E. cloacae* [38,39].

Many studies have indicated that patients infected with third generation or broad-spectrum cephalosporin resistant/nonsusceptible isolates, including *Enterobacter* spp. [19,40,41] had a worse clinical response, more days in hospital, a poorer outcome, and a higher mortality rate [19,42,43,44,45] than those infected with susceptible isolates. Our study found that patients infected with third generation cephalosporin-resistant *Enterobacter* had higher 30-day and 100-day mortality rates than those infected with third generation cephalosporin susceptible/intermediate *Enterobacter*, though patients with *Enterobacter* resistant to third generation cephalosporins also had higher rates of kidney disease, indwelling devices use, and ICU admission.

In this study, we did not observe a significant difference between *E. hormaechei* and the other species in terms of demographic data, comorbidities, therapeutic devices and procedures, and clinical outcomes (30-day and 100-day mortality). With regard to the virulence of specific clusters, Liu et al. reported the virulence of cluster I strains was significantly higher than that of the other cluster strains according to the results of the *Galleria mellonella* infection model [46]. Cluster II (*E. kobei*) has strong biofilm formation ability under nutrient-deficient conditions but is associated with low virulence and pathogenicity [46]. However, the case number of cluster I in our study is too small to obtain enough clinical finding. Interestingly, we found the mortality rate to be zero for 18 cluster II patients in the study. Patients with *E. cloacae* subsp. *Cloacae* (cluster XI) had poor outcomes and had significantly higher 30-day mortality and 100-day mortality rates. The above suggests the *Enterobacter* species/cluster may have different clinical significance. However, the resistance to third generation cephalosporins clearly impacts the clinical outcome for *Enterobacter* infection.

Limitations of the research included (1) the fact that the *bla*_ACT_ gene was not detected in some species, which might be due to the variations of nucleotides at the primer sequences for intrinsic and plasmid-mediated AmpC β-lactamase genes, subsequently leading to missed detection using PCR. Further research is needed. (2) We aimed to investigate the clinical and microbiological characteristics of the species within *E. cloacae* complex (ECC) in this research. Our bacteria material was ECC isolates which were identified using an automated system in a clinical laboratory, but this did not include all *Enterobacter* species. Therefore, the research findings apply to species in ECC but not all *Enterobacter* species.

## 4. Materials and Methods

### 4.1. Bacterial Isolates

A total of 184 consecutive *Enterobacter* isolates identified as *E. cloacae* complex with a VITEK 2 system were collected from Kaohsiung Medical University Hospital (KMUH), a 1720-bed medical center in Kaohsiung, Taiwan, from December 1, 2013, to June 14, 2014. The identification of bacterial isolates was performed using the VITEK 2 microbial identification system (bioMérieux, Hazelwood, MO, USA). Isolates were stored at −80 °C in GermBank stocks (CMP^TM^ Culture Media, New Taipei City, Taiwan) until processing.

### 4.2. Antimicrobial Susceptibility Testing

Antimicrobial susceptibility was tested using the broth dilution method according to the guidelines of the Clinical and Laboratory Standards Institute (CLSI) [47]. The following antimicrobial agents were tested: ampicillin, amikacin, ceftazidime, cefmetazole, ceftriaxone, cefazolin, ertapenem, cefepime, gentamicin, levofloxacin, meropenem, ampicillin/sulbactam, sulfamethoxazole/trimethoprim, tigecycline, and piperacillin/tazobactam. Isolates resistant to at least one antimicrobial agent in three or more antimicrobial classes are defined as multidrug resistant isolates.

### 4.3. Species Identification of ECC Based on hsp60 Sequencing

Polymerase chain reaction (PCR) analysis for partial sequencing of the *hsp60* gene was performed using primers; the conditions and protocol were as described previously [8]. A 341-bp fragment of the *hsp60* gene was amplified and sequenced. A 272-bp fragment of the *hsp60* gene was obtained for the 184 strains, and its sequence was analyzed using BLAST searches on the NCBI website against nucleotide databases. Sequences were analyzed using MEGA 11 software (version 11.0.13). The sequence of the fragment was compared to reference sequences from type strains previously described in taxonomic studies [3,8] using the ClustalW algorithm. The type strains were described previously [4,5,6,8,10]. The phylogenetic tree was constructed using neighbor-joining analysis. Thus, each isolate was assigned to its respective species and cluster.

### 4.4. Detection of Genes Encoding ESBLs, AmpC, and Carbapenemases

PCR was used to detect the genes encoding ESBLs (CTX-M, SHV, and TEM) [48,49,50], AmpC (CMY, DHA, MIR, and ACT) [51,52,53], and carbapenemases (IMP, KPC, OXA, NDM, VIM, BIC, IMI, SME, AIM, DIM, GIM, SPM, SIM, and GES) [54]. Amplicons were sequenced to determine the genotypes of various β-lactamase genes.

### 4.5. Analysis of Class 1 Integrons and Gene Cassettes

PCR was used to detect the presence of class 1 integrons and to amplify class 1 integron cassettes as previously described [55,56]. Gene cassettes within the class 1 integrons were identified using nucleotide sequencing, and similarity searches of each gene with nucleotide sequences in the GenBank database were performed with the BLASTN program (https://blast.ncbi.nlm.nih.gov/Blast.cgi, accessed on 17 March 2022).

### 4.6. Pulsed Field Gel Electrophoresis (PFGE)

Clonal relatedness of *Enterobacter* isolates was determined using PFGE, which was performed according to a previously described protocol [57]. The restriction enzyme *Xba*I (New England Biolabs Inc., MA, USA) was used at the temperature suggested by the manufacturer. Restriction fragments were analyzed using GelCompar II software 6.5 (Applied Maths, Austin, TX, USA), and dendrograms of the patterns were constructed using the unweighted pair group method with the arithmetic mean based on the Dice similarity index. PFGE patterns were interpreted in accordance with the criteria of Tenover et al. [58]. Isolates with >85% similarity in PFGE banding patterns were designated as a pulsotype.

### 4.7. Analysis of Clinical Features of Patients Infected with ECC

This was a retrospective, observational study of patients with positive cultures of ECC from 1 December 2013, to 14 June 2014, at KUMH. Patients who underwent repeated sampling within 2 months, those infected with microorganisms other than *Enterobacter*, and those with incomplete medical records were excluded. A total of 161 patients were analyzed. Patient information was retrospectively retrieved from electronic medical records. The parameters included demographic data, comorbidities, therapeutic devices, and procedures (such as indwelling devices, hemodialysis, mechanical ventilation, and surgeries), exposure to drugs prior to isolation (steroids within 3 months, antimicrobials within 3 months and 2 weeks), sites of acquisition, and clinical outcomes. Sites of acquisition included hospital-acquired, community-acquired and healthcare-associated infections. Hospital-acquired infection was defined as an infection that occurred >48 h after admission to the hospital [19]. Community-acquired infection was defined as infection in patients undergoing outpatient treatment who had not been hospitalized or had not resided in a healthcare facility in the previous 3 months [44,59]. Healthcare-associated infection was defined as patients undergoing outpatient treatment who had been hospitalized or had resided in a healthcare facility in the previous 3 months. Clinical outcomes were assessed based on 30-day mortality or 100-day mortality from specimen collection.

### 4.8. Statistical Analyses

The chi-square test or Fisher exact test was used to compare categorical variables. Statistical significance was set at *p* < 0.05. All statistical analyses were performed using IBM SPSS AMOS 20.0 software.

## 5. Conclusions

In conclusion, third generation cephalosporin resistance, multidrug resistance and class 1 integrons are significantly higher in *E. hormaechei* (clusters III, VI, VII, and VIII), compared to the other species/clusters. Patients infected with third generation cephalosporin-resistant *Enterobacter* have significantly higher 30-day mortality and 100-day mortality rates than those infected with *Enterobacter* susceptible/intermediate to third generation cephalosporins. Our findings on the unequal distribution of drug resistance profiles and class 1 integrons among *Enterobacter* species/cluster and the significant clinical impact of some species further emphasize the need for a larger scale investigation of the species of *Enterobacter*.

## Figures and Tables

**Table 1:** Species identification using *hsp60* gene sequencing of *Enterobacter* isolates.

Species	Cluster	*n*	%	Nomenclature of Species by Wu et al. [4]
*Enterobacter asburiae*	I	4	2.2	*Enterobacter asburiae*
*Enterobacter kobei*	II	19	10.3	*Enterobacter kobei*
*Enterobacter hormaechei* subsp. *hoffmannii*	III	10	5.4	*Enterobacter hoffmannii*
*Enterobacter roggenkampii*	IV	13	7.1	*Enterobacter roggenkampii*
*Enterobacter hormaechei* subsp. *oharae*	VI	37	20.1	*Enterobacter xiangfangensis* ^a^
*Enterobacter hormaechei* subsp. *hormaechei*	VII	3	1.6	*Enterobacter hormaechei*
*Enterobacter hormaechei* subsp. *steigerwaltii*	VIII	55	29.9	*Enterobacter xiangfangensis* ^a^
*Enterobacter bugandensis*	IX	9	4.9	*Enterobacter bugandensis*
*Enterobacter cloacae* subsp. *cloacae*	XI	22	12.0	*Enterobacter cloacae*
*Enterobacter cloacae* subsp. *dissolvens*	XII	2	1.1	*Enterobacter dissolvens*
*Enterobacter chuandaensis*	-	1	0.5	*Enterobacter chuandaensis*
*Enterobacter mori*	-	2	1.1	*Enterobacter mori*
*Enterobacter quasihormaechei*	-	2	1.1	*Enterobacter quasihormaechei*
*Enterobacter sichuanensis*	-	1	0.5	*Enterobacter sichuanensis*
Not determined	-	4	2.2	-

^a^*Enterobacter xiangfangensis* are composed of *Enterobacter hormaechei* subsp. *oharae* (cluster VI) and *Enterobacter hormaechei* subsp. *steigerwaltii* (cluster VIII) according to Wu’s classification [4].

**Table 2:** Antimicrobial resistance rates in each species of *Enterobacter*.

Species (*n*)	Cluster	AN		CAZ		CRO		ETP		FEP		GM		LFV		MEM		SXT		TGC		TZP	
		*n*	%	*n*	%	*n*	%	*n*	%	*n*	%	*n*	%	*n*	%	*n*	%	*n*	%	*n*	%	*n*	%
*E. asburiae* (4)	I	0	0.0	0	0.0	0	0.0	0	0.0	0	0.0	0	0.0	0	0.0	0	0.0	0	0.0	0	0.0	0	0.0
*E. kobei* (19)	II	0	0.0	3	15.8	3	15.8	0	0.0	0	0.0	0	0.0	0	0.0	0	0.0	1	5.3	0	0.0	3	15.8
*E.hormaechei* subsp. *hoffmannii* (10)	III	0	0.0	3	30.0	5	50.0	1	10.0	0	0.0	2	20.0	6	60.0	0	0.0	9	90.0	6	60.0	4	40.0
*E.roggenkampii* (13)	IV	0	0.0	2	15.4	3	23.1	0	0.0	0	0.0	0	0.0	0	0.0	0	0.0	0	0.0	1	7.7	3	23.1
*E. hormaechei* subsp. *oharae* (37)	VI	0	0.0	15	40.5	15	40.5	2	5.4	0	0.0	7	18.9	3	8.1	0	0.0	14	37.8	1	2.7	12	32.4
*E. hormaechei* subsp. *hormaechei* (3)	VII	0	0.0	1	33.3	0	0.0	0	0.0	0	0.0	1	33.3	1	33.3	0	0.0	1	33.3	0	0.0	0	0.0
*E. hormaechei* subsp. *steigerwaltii* (55)	VIII	0	0.0	17	30.9	19	34.5	5	9.1	2	3.6	3	5.5	2	3.6	1	1.8	7	12.7	6	10.9	13	23.6
*E.bugandensis* (9)	IX	0	0.0	1	11.1	1	11.1	0	0.0	0	0.0	0	0.0	0	0	0	0.0	0	0.0	0	0.0	1	11.1
*E. cloacae* subsp. *cloacae* (22)	XI	0	0.0	6	27.3	6	27.3	0	0.0	0	0.0	0	0.0	0	0.0	0	0.0	2	9.1	0	0.0	6	27.3
*E. cloacae* subsp. *dissolvens* (2)	XII	0	0.0	0	0.0	0	0.0	0	0.0	0	0.0	0	0.0	0	0.0	0	0.0	0	0.0	0	0.0	0	0.0
*E. chuandaensis* (1)	-	0	0.0	0	0.0	0	0.0	0	0.0	0	0.0	0	0.0	1	100	0	0.0	0	0.0	0	0.0	0	0.0
*E. mori* (2)	-	0	0.0	0	0.0	0	0.0	0	0.0	0	0.0	0	0.0	0	0.0	0	0.0	0	0.0	0	0.0	0	0.0
*E. quasihormaechei* (2)	-	0	0.0	0	0.0	0	0.0	0	0.0	0	0.0	0	0.0	0	0.0	0	0.0	0	0.0	0	0.0	0	0.0
*E. sichuanensis*(1)	-	0	0.0	1	100	1	100	0	0.0	0	0.0	0	0.0	0	0.0	0	0.0	0	0.0	0	0.0	1	100
Not determined (4)	-	0	0.0	1	25.0	1	25.0	0	0.0	0	0.0	0	0.0	0	0.0	0	0.0	0	0.0	0	0.0	1	25.0
Total (184)		0	0.0	50	27.2	54	29.3	8	4.3	2	1.1	13	7.1	13	7.1	1	0.5	34	18.5	14	7.6	44	23.9

AN, amikacin; CAZ, ceftazidime; CRO, ceftriaxone; ETP, ertapenem; FEP, cefepime; GM, gentamicin; LFV, levofloxacin; MEM, meropenem; SXT, sulfamethoxazole/trimethoprim; TGC, tigecycline; TZP, piperacillin/tazobactam.

**Table 3:** Distribution of β-lactamase genes in species of *Enterobacter*.

Species (*n*)	Cluster	β-Lactamase Genes (*n*)				
		*bla*_ESBL_	*bla*_AmpC_	*bla* Genes Encoding Carbapenemases	Coexistence of *bla*_ESBL_ and *bla*_AmpC_ Genes	Other β-Lactamase Genes
*E. asburiae* (4)	I					
*E. kobei* (19)	II		*bla*_ACT_ (1)			
*E. hormaechei* subsp. *hoffmannii* (10)	III		*bla*_ACT_ (5) *bla*_ACT_+*bla*_DHA-1_ (3)			*bla*_TEM-1_ (3) *bla*_OXA-1_+*bla*_TEM-1_ (1)
*E.roggenkampii* (13)	IV		*bla*_MIR_ (3)			
*E. hormaechei* subsp. *oharae* (37)	VI	*bla*_SHV-12_ (1)	*bla*_ACT_ (5)		*bla*_SHV-12_+*bla*_ACT_+*bla*_DHA-1_ (1)	*bla*_TEM-1_ (5)
*E. hormaechei* subsp. *hormaechei* (3)	VII				*bla*_SHV-12_+*bla*_ACT_+*bla*_DHA-1_ (1)	*bla*_TEM-1_ (1)
*E. hormaechei* subsp. *steigerwaltii* (55)	VIII	*bla*_CTX-M-15_ (1)	*bla*_ACT_ (2) *bla*_DHA-1_ (1)	*bla*_IMP-8_ (1)		*bla*_TEM-1_ (4) *bla*_OXA-1_ (2)
*E. bugandensis* (9)	IX		*bla*_ACT_ (1)			
*E. cloacae* subsp. *cloacae* (22)	XI	*bla*_SHV-12_ (1)				*bla*_TEM-1_ (1)
*E. cloacae* subsp. *dissolvens* (2)	XII					
*E. chuandaensis* (1)	-					*bla*_TEM-1_ (1)
*E. mori* (2)	-					
*E. quasihormaechei* (2)	-					
*E. sichuanensis*(1)	-					
Not determined (4)	-		*bla*_ACT_ (1)			
Total (184)		3	22	1	2	18

**Table 4:** Class 1 integrons and their gene cassettes in species of *Enterobacter*.

Species (*n*)	Cluster	*intI1* (+) *n*	Gene Cassette Array of Class 1 Integrons (*n*)
*E. asburiae* (4)	I	-	-
*E. kobei* (19)	II	2	*aadA2* (2)
*E. hormaechei* subsp. *hoffmannii* (10)	III	10	*dfrA15* (6) *aadA2* (2) *aac(6′)-IIc-ereA2* (pseudogene)*-*IS*1247-aac3-arr-ereA2* (pseudogene) (1) *aadB/aadA2* (1) ^a^
*E.roggenkampii* (13)	IV	2	*aadA2* (1)
*E. hormaechei* subsp. *oharae* (37)	VI	16	*aadB* (1) *aadA2* (2) *aadB-aadA2* (2) *dfrA12-orfF-aadA2* (3) *aac(6′)-IIc-ereA2* (pseudogene)*-*IS*1247-aac3-arr-ereA2* (pseudogene) (1) *aadB/aadA2* (2) ^a^ *aadA2/aadB-aadA2/aadA2-aadA2* (1) ^b^
*E. hormaechei* subsp. *hormaechei* (3)	VII	1	*aadA2* (1)
*E. hormaechei* subsp. *steigerwaltii* (55)	VIII	9	*aadA1* (2) *dfrA7/aadA2* (1) ^a^
*E. bugandensis* (9)	IX	-	-
*E. cloacae* subsp. *cloacae* (22)	XI	4	*aadA2* (1) *aac(6′)-Ib-cr-arr3-dfrA27* (1)
*E. cloacae* subsp. *dissolvens* (2)	XII	-	-
*E. chuandaensis* (1)	-	1	*dfrA12-orfF-aadA2* (1)
*E. mori* (2)	-	-	-
*E. quasihormaechei* (2)	-	-	-
*E. sichuanensis*(1)	-	-	-
Not determined (4)	-	-	-
Total (184)		45 (24.5%)	32

^a^ These four isolates carry two class 1 integrons; ^b^ this isolate carries three class 1 integrons.

**Table 5:** Clinical characteristics and outcomes of patients infected with *Enterobacter* resistant to third generation cephalosporins.

Parameter (*n* = 161)	Infected with the Third Generation Cephalosporin Resistant *Enterobacter* *n* = 49 (%)	Infected with the Third Generation Cephalosporin Susceptible/Intermediate *Enterobacter* *n* = 112 (%)	χ^2^	*p* Value ^a^	OR (95% CI)
Age (years)					
18–65	20 (40.8)	60 (53.6)	2.22	0.136	0.60 (0.30–1.18)
>65	29 (59.2)	52 (46.4)	2.22	0.136	1.67 (0.85–3.30)
Sex					
Male	34 (69.4)	72 (64.3)	0.39	0.532	1.26 (0.61–2.59)
Female	15 (30.6)	40 (35.7)	0.39	0.532	0.79 (0.39–1.63)
Location					
Outpatient	4 (8.2)	26 (23.2)	5.09	**0.024**	0.29 (0.10–0.89)
Ward	45 (91.8)	86 (76.8)	5.09	**0.024**	3.40 (1.12–10.35)
Isolation specimens					
Ascites	1 (2.0)	0 (0.0)	NA	NA	NA
Blood	8 (16.3)	18 (16.1)	0	1	1.02 (0.41–2.53)
Sputum	16 (32.7)	26 (23.2)	1.57	0.210	1.60 (0.76–3.36)
Bile	4 (8.2)	12 (10.7)	NA	0.778	0.74 (0.23–2.42)
Urine	16 (32.7)	30 (26.8)	0.57	0.450	1.33 (0.64–2.75)
Abscess/Pus	4 (8.2)	26 (23.2)	5.09	**0.024**	0.29 (0.10–0.89)
Comorbidities					
Diabetes mellitus	22 (44.9)	39 (34.8)	1.47	0.225	1.53 (0.77–3.02)
Hypertension	25 (51.0)	55 (49.1)	0.05	0.823	1.08 (0.55–2.11)
Kidney disease	29 (59.2)	41 (36.6)	7.07	**0.007**	2.51 (1.26–4.99)
Gastrointestinal disease	15 (30.6)	26 (23.2)	0.98	0.322	1.46 (0.69–3.09)
Urinary tract infection	24 (49.0)	38 (33.9)	3.26	0.071	1.87 (0.94–3.70)
Heart failure	5 (10.2)	18 (16.1)	0.96	0.327	0.59 (0.21–1.70)
Cerebrovascular disease	10 (20.4)	18 (16.1)	0.45	0.502	1.34 (0.57–3.16)
Pulmonary disease	23 (46.9)	46 (41.1)	0.48	0.488	1.27 (0.65–2.49)
Malignancy	13 (26.5)	30 (26.8)	0	1	0.99 (0.46–2.11)
Drug exposure					
Steroid exposure in the past 3 months	19 (38.8)	43 (38.4)	0	1	1.02 (0.51–2.02)
Antibiotics exposure in the past 3 months	47 (95.9)	103 (92.0)	NA	0.506	2.05 (0.43–9.88)
Antibiotic exposure in the past 2 weeks	42 (85.7)	96 (85.7)	0	1	1 (0.38–2.61)
Therapeutic devices and procedures in the past 3 months					
Hemodialysis	7 (14.3)	9 (8.0)	6.02	0.256	1.91 (0.67–5.46)
Chemotherapy	7 (14.3)	16 (14.3)	0	1	1 (0.38–2.61)
Indwelling devices	48 (98.0)	98 (87.5)	NA	**0.040**	6.86 (0.88–53.69)
Transplantation	0 (0.0)	4 (3.6)	NA	0.315	NA
Surgery	27 (55.1)	48 (42.9)	2.05	0.152	1.64 (0.83–3.22)
Site of acquisition					
Hospital-acquired	31 (63.3)	54 (48.2)	3.1	0.078	1.85 (0.93–3.68)
Community-acquired	5 (10.2)	20 (17.9)	1.52	0.218	0.52 (0.18–1.48)
Healthcare-associated	13 (26.5)	38 (33.9)	0.86	0.354	0.70 (0.33–1.48)
ICU admission	25 (51.0)	32 (28.6)	7.51	**0.006**	2.60 (1.30–5.21)
Class 1 integron	21 (42.9)	18 1(6.1)	13.32	**<0.001**	3.92 (1.84–8.36)
Outcomes					
30-day mortality	14 (28.6)	7 (6.3)	14.97	**<0.001**	6 (2.24–16.06)
100-day mortality	15 (30.6)	8 (7.1)	15.33	**<0.001**	5.74 (2.24–14.70)

^a^*p* < 0.05 indicated statistical significance and these values are presented in boldface. NA, not available.

## Data Availability

Data sharing not applicable.

## Supplementary Materials

The following are available online at https://www.mdpi.com/article/10.3390/antibiotics11091153/s1, File S1: Figure S1. The phylogenetic tree resulting from analysis of the *hsp60* gene sequences of 184 *Enterobacter* isolates and previously reported sequences of type strains. File S2: The partial *hsp60* sequences of 184 *Enterobacter* isolates. File S3: Table S1. Clinical characteristics and outcomes of cases infected with *E. hormaechei* (clusters III, VI, VII, and VIII) and the other *Enterobacter* species, Table S2. Clinical characteristics and outcomes of cases infected with the four most common species/clusters in this study, Table S3. Comparison of the distribution of *Enterobacter* species among ECC in different countries.
