# Supplementary material for: The Resistance Mechanisms and Clinical Impact of Resistance to the Third Generation Cephalosporins in Species of Enterobacter cloacae Complex in Taiwan
Source: Antibiotics (Basel). 2022 Aug 26;11(9):1153. doi: 10.3390/antibiotics11091153 (PMC9494969; doi:10.3390/antibiotics11091153)
Supplement: Supplementary file 1 [file antibiotics-11-01153-s001.zip › Final proofreading-Supplementary File S1 Figure S1.pptx]

## Slide 1
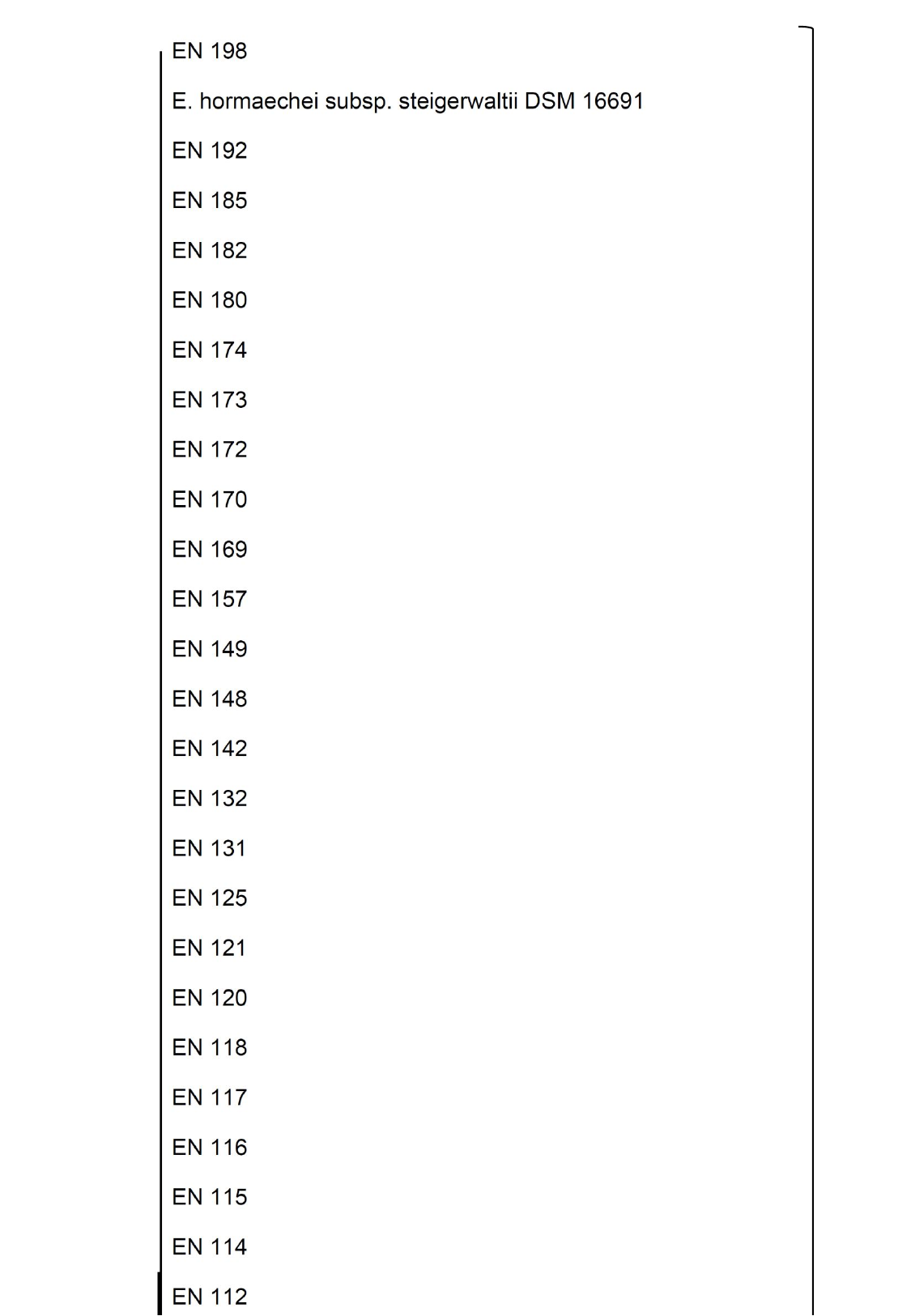

VIII
VII
VI
IX
XI
IV
XII
I
III
II

## Slide 2
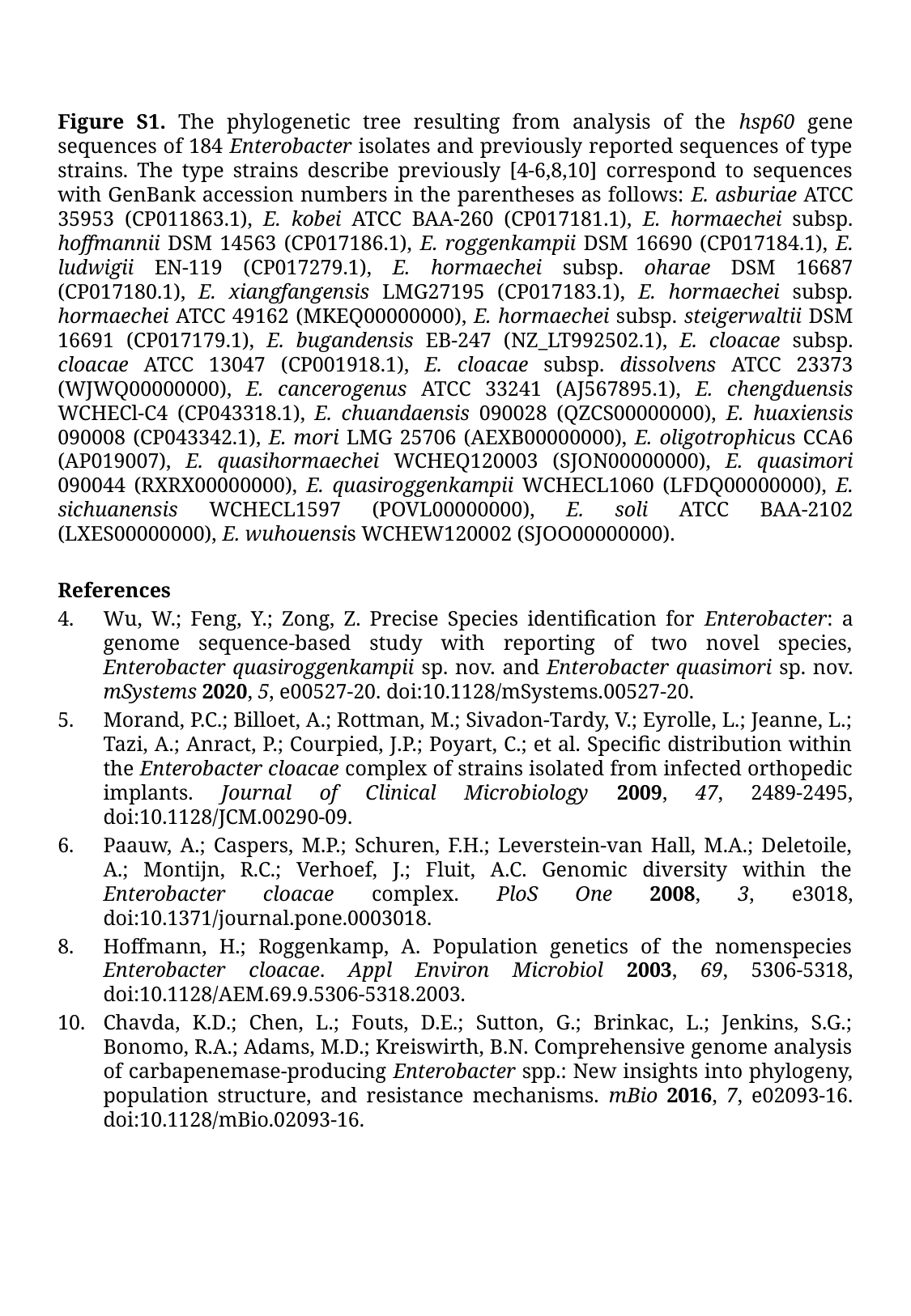

Figure S1. The phylogenetic tree resulting from analysis of the hsp60 gene sequences of 184 Enterobacter isolates and previously reported sequences of type strains. The type strains describe previously [4-6,8,10] correspond to sequences with GenBank accession numbers in the parentheses as follows: E. asburiae ATCC 35953 (CP011863.1), E. kobei ATCC BAA-260 (CP017181.1), E. hormaechei subsp. hoffmannii DSM 14563 (CP017186.1), E. roggenkampii DSM 16690 (CP017184.1), E. ludwigii EN-119 (CP017279.1), E. hormaechei subsp. oharae DSM 16687 (CP017180.1), E. xiangfangensis LMG27195 (CP017183.1), E. hormaechei subsp. hormaechei ATCC 49162 (MKEQ00000000), E. hormaechei subsp. steigerwaltii DSM 16691 (CP017179.1), E. bugandensis EB-247 (NZ_LT992502.1), E. cloacae subsp. cloacae ATCC 13047 (CP001918.1), E. cloacae subsp. dissolvens ATCC 23373 (WJWQ00000000), E. cancerogenus ATCC 33241 (AJ567895.1), E. chengduensis WCHECl-C4 (CP043318.1), E. chuandaensis 090028 (QZCS00000000), E. huaxiensis 090008 (CP043342.1), E. mori LMG 25706 (AEXB00000000), E. oligotrophicus CCA6 (AP019007), E. quasihormaechei WCHEQ120003 (SJON00000000), E. quasimori 090044 (RXRX00000000), E. quasiroggenkampii WCHECL1060 (LFDQ00000000), E. sichuanensis WCHECL1597 (POVL00000000), E. soli ATCC BAA-2102 (LXES00000000), E. wuhouensis WCHEW120002 (SJOO00000000).
References
Wu, W.; Feng, Y.; Zong, Z. Precise Species identification for Enterobacter: a genome sequence-based study with reporting of two novel species, Enterobacter quasiroggenkampii sp. nov. and Enterobacter quasimori sp. nov. mSystems 2020, 5, e00527-20. doi:10.1128/mSystems.00527-20.
Morand, P.C.; Billoet, A.; Rottman, M.; Sivadon-Tardy, V.; Eyrolle, L.; Jeanne, L.; Tazi, A.; Anract, P.; Courpied, J.P.; Poyart, C.; et al. Specific distribution within the Enterobacter cloacae complex of strains isolated from infected orthopedic implants. Journal of Clinical Microbiology 2009, 47, 2489-2495, doi:10.1128/JCM.00290-09.
Paauw, A.; Caspers, M.P.; Schuren, F.H.; Leverstein-van Hall, M.A.; Deletoile, A.; Montijn, R.C.; Verhoef, J.; Fluit, A.C. Genomic diversity within the Enterobacter cloacae complex. PloS One 2008, 3, e3018, doi:10.1371/journal.pone.0003018.
Hoffmann, H.; Roggenkamp, A. Population genetics of the nomenspecies Enterobacter cloacae. Appl Environ Microbiol 2003, 69, 5306-5318, doi:10.1128/AEM.69.9.5306-5318.2003.
Chavda, K.D.; Chen, L.; Fouts, D.E.; Sutton, G.; Brinkac, L.; Jenkins, S.G.; Bonomo, R.A.; Adams, M.D.; Kreiswirth, B.N. Comprehensive genome analysis of carbapenemase-producing Enterobacter spp.: New insights into phylogeny, population structure, and resistance mechanisms. mBio 2016, 7, e02093-16. doi:10.1128/mBio.02093-16.
