# Supplementary material for: The Resistance Mechanisms and Clinical Impact of Resistance to the Third Generation Cephalosporins in Species of Enterobacter cloacae Complex in Taiwan
Source: Antibiotics (Basel). 2022 Aug 26;11(9):1153. doi: 10.3390/antibiotics11091153 (PMC9494969; doi:10.3390/antibiotics11091153)
Supplement: Supplementary file 1 [file antibiotics-11-01153-s001.zip › Final proofreading-Supplementary File S3 Table S1-S3.pdf]

Table S1. Clinical characteristics and outcomes of cases infected with *E. hormaechei* (clusters III, VI, VII, and VIII) and the other *Enterobacter* species

**Parameter (*n* = 161)**	**Infected with *E. hormaechei* *n* = 91 (%)**	**Infected with the other *Enterobacter* species *n* = 70 (%)**	χ^2	***P* value[a]**	**OR (95% CI)**
Age (years)					
18–65	42 (46.2)	38 (54.3)	1.05	0.306	0.72 (0.39-1.35)
>65	49 (53.8)	32 (45.7)	1.05	0.306	1.39 (0.74-2.59)
Sex					
Male	64 (70.3)	42 (60.0)	1.88	0.170	1.58 (0.82-3.05)
Female	27 (29.7)	28 (40.0)	1.88	0.170	0.63 (0.33-1.22)
Location					
Outpatient	16 (17.6)	14 (20.0)	0.15	0.699	0.85 (0.38-1.89)
Ward	75 (82.4)	56 (80.0)	0.15	0.699	1.17 (0.53-2.60)
Isolation specimens					
Ascites	1 (1.1)	0	NA	NA	NA
Blood	15 (16.5)	11 (15.7)	0.02	0.888	1.06 (0.45-2.47)
Sputum	21 (23.1)	21 (30.0)	0.98	0.322	0.7 (0.35-1.42)
Bile	7 (7.7)	9 (12.9)	1.18	0.277	0.56 (0.20-1.60)
Urine	30 (33.0)	16 (22.9)	1.98	0.159	1.66 (0.82-3.37)
Abscess/Pus	17 (18.7)	13 (18.6)	0	1	1.01 (0.45-2.24)
Comorbidities					
Diabetes mellitus	32 (35.2)	29 (41.4)	0.66	0.417	0.77 (0.40-1.46)
Hypertension	42 (46.2)	38 (54.3)	1.05	0.306	0.72 (0.39-1.35)
Kidney disease	42 (46.2)	28 (40.0)	0.61	0.435	1.29 (0.68-2.42)
Gastrointestinal disease	19 (20.9)	22 (31.4)	2.32	0.128	0.5 8 (0.28-1.18)
Urinary tract infection	32 (35.2)	30 (42.9)	0.99	0.320	0.72 (0.38-1.37)
Heart failure	12 (13.2)	11 (15.7)	0.21	0.647	0.81 (0.34-1.97)
Cerebrovascular disease	18 (19.8)	10 (14.3)	0.83	0.362	1.48 (0.64-3.44)
Pulmonary disease	39 (42.9)	30 (42.9)	0	1	1 (0.53-1.88)
Malignancy	22 (24.2)	21 (30.0)	0.69	0.406	0.74 (0.37-1.50)
Drug exposure					
Steroid exposure in the past 3 months	34 (37.4)	28 (40.0)	0.12	0.729	0.89 (0.47-1.70)
Antibiotics exposure in the past 3 months	83 (91.2)	67 (95.7)	NA	0.351	0.46 (0.12-1.82)
Antibiotics exposure in the	76 (83.5)	62 (88.6)	0.83	0.362	0.65 (0.26-1.64)

Parameter (n = 161)	Infected with *E. hormaechei* n = 91 (%)	Infected with the other *Enterobacter* species n = 70 (%)	χ^2	*P* value[a]	OR (95% CI)
past 2 weeks					
Therapeutic devices and procedures in the past 3 months					
Hemodialysis	10 (11.0)	6 (8.6)	0.26	0.610	1.32 (0.45-3.82)
Chemotherapy	12 (13.2)	11 (15.7)	0.21	0.647	0.81 (0.34-1.97)
Indwelling devices	81 (89.0)	65 (92.9)	0.69	0.406	0.62 (0.20-1.91)
Transplantation	1 (1.1)	3 (4.3)	NA	0.318	0.25 (0.03-2.44)
Surgery	35 (38.5)	40 (57.1)	5.55	**0.018**	0.47 (0.25-0.88)
Site of acquisition					
Hospital-acquired	52 (57.1)	33 (47.1)	1.59	0.207	1.49 (0.80-2.80)
Community-acquired	10 (11.0)	15 (21.4)	3.29	0.070	0.45 (0.19-1.08)
Healthcare-associated	29 (31.9)	37 (52.9)	7.21	**0.007**	0.42 (0.22-0.79)
ICU admission	38 (41.8)	22 (31.4)	1.81	0.179	1.56 (0.81- 3.01)
The third generation cephalosporin resistant	34 (37.4)	15 (21.4)	9.74	**<0.001**	14.66 (6.49-33.13)
Class 1 integron	30 (33.0)	9 (12.9)	8.72	**0.003**	3.33 (1.46-7.61)
Outcomes					
30-day mortality	13 (14.3)	8 (11.4)	0.28	0.60	1.29 (0.50-3.31)
100-day mortality	14 (15.4)	9 (12.9)	0.21	0.647	5.60 (2.14-14.65)

[a] *P* <0.05 indicated statistical significance and these values are presented in boldface.

NA, not available

Table S2. Clinical characteristics and outcomes of cases infected with the four most common species/clusters in this study

**Parameter** (*n* = 116)	**Infected with *E. kobei*** *n* = 18 (%)	**Infected with *E. hormaechei* subsp. *oharae*** *n* = 33 (%)	**Infected with *E. hormaechei* subsp. *steigerwaltii*** *n* = 47 (%)	**Infected with *E. cloacae* subsp. *cloacae*** *n* = 18 (%)	χ^2	***P* value**[a]
Age (years)					3.62	0.306
18–65	10 (55.6)	13 (39.4)	28 (59.6)	8 (44.4)		
>65	8 (44.4)	20 (60.6)	19 (40.4)	10 (55.6)		
Sex					6.86	0.076
Male	8 (44.4)	19 (57.6)	36 (76.6)	11 (61.1)		
Female	10 (55.6)	14 (42.4)	11 (23.4)	7 (38.9)		
Location					3.41	0.333
Outpatient	2 (11.1)	8 (24.2)	8 (17.0)	1 (5.6)		
Ward	16 (88.9)	25 (75.8)	39 (83.0)	17 (94.4)		
Isolation specimens						
Ascites	0	1 (3.0)	0	0	NA	NA
Blood	4 (22.2)	4 (12.1)	6 (12.8)	1 (5.6)	2.27	0.518
Sputum	5 (27.8)	7 (21.2)	12 (25.5)	8 (44.4)	3.33	0.343
Bile	3 (16.7)	2 (6.1)	3 (6.4)	2 (11.1)	2.19	0.533
Urine	3 (16.7)	11 (10.2)	17 (36.2)	5 (27.8)	2.49	0.478
Abscess/Pus	3 (16.7)	8 (24.2)	9 (19.1)	2 (11.1)	1.38	0.709
Comorbidities						
Diabetes mellitus	6 (33.3)	10 (30.3)	16 (34.0)	10 (55.6)	3.58	0.311
Hypertension	9 (50.0)	15 (45.5)	19 (40.4)	11 (61.1)	2.34	0.504
Kidney disease	5 (27.8)	15 (45.5)	19(40.4)	9 (50.0)	2.17	0.538
Gastrointestinal disease	6 (33.3)	8 (24.2)	6 (12.8)	8 (44.4)	8.20	**0.042**
Urinary tract infection	7 (38.9)	12 (36.4)	15(31.9)	9 (50.0)	1.86	0.602
Heart failure	3 (16.7)	5 (15.2)	5 (10.6)	2 (11.1)	0.64	0.887
Cerebrovascular disease	3 (16.7)	8 (24.2)	8 (17.0)	4 (22.2)	0.82	0.846
Pulmonary disease	8 (44.4)	15 (45.5)	15 (31.9)	9 (50.0)	2.56	0.464
Malignancy	6 (33.3)	6 (18.2)	14 (29.8)	4 (22.2)	2.04	0.564
Drug exposure						
Steroid use in the past 3 months	7 (38.9)	14 (42.4)	17 (36.2)	8 (44.4)	0.52	0.914

Parameter (*n* = 116)	Infected with *E. kobei* *n* = 18 (%)	Infected with *E. hormaechei* subsp. *oharae* *n* = 33 (%)	Infected with *E. hormaechei* subsp. *steigerwaltii* *n* = 47 (%)	Infected with *E. cloacae* subsp. *cloacae* *n* = 18 (%)	χ^2	*P* value[a]
Antibiotic use in the past 3 months	18 (100)	32 (97.0)	40 (85.1)	17 (94.4)	6.01	0.111
Antibiotic use in the past 2 weeks	17 (94.4)	28 (84.8)	37 (78.7)	17 (94.4)	4.04	0.257
Therapeutic devices used and procedures undertaken in the past 3 months						
Hemodialysis	2 (11.1)	5 (15.2)	0	0	9.83	**0.020**
Chemotherapy	2 (11.1)	4 (12.1)	8 (17.0)	3 (16.7)	0.62	0.892
Indwelling devices	18 (100)	28 (84.8)	42 (89.4)	17 (94.4)	3.52	0.318
Transplantation	0	1(3.0)	0	1 (5.6)	3.03	0.387
Surgery	9(50.0)	13(39.4)	16(34.0)	12 (66.7)	6.18	0.103
Site of acquisition						
Hospital-acquired [b]	15 (83.3)	31 (93.9)	41 (87.2)	11 (61.1)	10.04	**0.018**
Community-acquired	3 (16.7)	2 (6.1)	6 (12.8)	7 (38.9)	10.04	**0.018**
ICU admission	2 (11.1)	13 (39.4)	17 (36.2)	9 (50.0)	6.57	0.087
The third generation cephalosporin resistant	2 (11.1)	14 (42.4)	14 (29.8)	7 (38.9)	5.77	0.124
β-lactamase	1 (5.6)	9 (27.3)	5 (10.6)	1 (5.6)	7.49	0.058
Class 1 integron	2 (11.1)	14 (42.4)	6 (12.8)	4 (22.2)	11.44	**0.010**
Outcomes						
30-day mortality	0	6 (18.2)	4 (8.5)	6 (33.3)	10.30	**0.016**
100-day mortality	0	7 (21.2)	4 (8.5)	6 (33.3)	10.67	**0.014**

[a] $P < 0.05$ indicated statistical significance and these values are presented in boldface.

[b] Healthcare-associated infections were included.

NA, not available

Table S3. Comparison of the distribution of *Enterobacter* species among ECC in different countries

Country	Species (Cluster)				Reference
	***E. hormaechei* subsp. *steigerwaltii* (VIII)**	***E. hormaechei* subsp. *hoffmannii* (III)**	***E. hormaechei* subsp. *oharae* (VI)**	***E. ludwigii* (V)**	
Germany	31%	25%	9%	9%	[26]
France	32.7%	42%	15.4%		[5]
Netherlands	49.4%	16.5%	8.2%		[6]
Taiwan	29.9%	5.4%	20.1%		This study

References

5. Morand, P.C.; Billoet, A.; Rottman, M.; Sivadon-Tardy, V.; Eyrolle, L.; Jeanne, L.; Tazi, A.; Anract, P.; Courpied, J.P.; Poyart, C.; et al. Specific distribution within the *Enterobacter cloacae* complex of strains isolated from infected orthopedic implants. *Journal of Clinical Microbiology* **2009**, *47*, 2489-2495, doi:10.1128/JCM.00290-09.
6. Paauw, A.; Caspers, M.P.; Schuren, F.H.; Leverstein-van Hall, M.A.; Deletoile, A.; Montijn, R.C.; Verhoef, J.; Fluit, A.C. Genomic diversity within the *Enterobacter cloacae* complex. *PloS One* **2008**, *3*, e3018, doi:10.1371/journal.pone.0003018.
26. Kremer, A.; Hoffmann, H. Prevalences of the *Enterobacter cloacae* complex and its phylogenetic derivatives in the nosocomial environment. *Eur J Clin Microbiol Infect Dis* **2012**, *31*, 2951-2955, doi:10.1007/s10096-012-1646-2.
